# Complex decongestive therapy and phlebolymphedema: Content of the treatment and measurement of limb volume

**DOI:** 10.1016/j.jvsv.2024.101840

**Published:** 2024-06-18

**Authors:** Musa Baklaci, Sibel Eyigor

**Affiliations:** Department of Physical Medicine and Rehabilitation, Kemalpaşa State Hospital, Izmir, Turkey; Department of Physical Medicine and Rehabilitation, School of Medicine, Ege University, Izmir, Turkey

We read with great pleasure the study titled "The beneficial role of complex decongestive therapy in patients with symptomatic chronic iliofemoral venous obstruction with phlebolymphedema" by Jayaraj and Thaggard.^1^ It is a valuable study on an essential topic in the literature. Thank you very much for your contributions to the literature. Despite the importance of the study, we would like to draw attention to some clinical issues.

The most commonly used treatment method for lymphedema is complete decongestive therapy (CDT), which includes patient education, skin care, exercises, manual lymphatic drainage (self), and compression therapy. In the study, patients receiving CDT consists of manual lymphatic drainage for 6 to 8 weeks and a lymphedema pump in conjunction with compression stockings or compression wraps. Failure to inform patients about patient education, skin care, and exercises results in incomplete CDT; as a result, patients' responses to treatment decreases. Additionally, there needs to be more information regarding the duration and pressure at which lymphedema pump therapy is administered to patients, and information about whether the CDT protocol was followed or compression stockings were used during the follow-up period would also be useful to the researcher.^2^^,^^3^

The study evaluated lymphedema by circumferential measurements of the knee, upper calf, midcalf, malleolus, and midfoot. The recommended method for determining the severity of lymphedema is the measurement of the total volume of the extremity. Extremity circumference measurements and volume calculations are the preferred methods because they are easy to apply. The deficiency in the data volume of the extremity causes an inadequate assessment of the level of lymphedema. When evaluating patients' responses to treatment, using gold standard measurement methods will be more helpful in evaluating the effectiveness of the treatment.^4^^,^^5^

CDT treatment seems to be effective in patients with symptomatic chronic iliofemoral venous obstruction with phlebolymphedema. However, the points mentioned in this letter should be taken into consideration in the future.

## Disclosures

None.
